# The effects of COVID-19 on Central Florida’s community gardens: lessons for promoting food security and overall community wellbeing

**DOI:** 10.3389/fpubh.2023.1147967

**Published:** 2023-07-06

**Authors:** William D. Schanbacher, James C. Cavendish

**Affiliations:** ^1^Department of Religious Studies, University of South Florida, Tampa, FL, United States; ^2^Department of Sociology and Interdisciplinary Social Sciences, University of South Florida, Tampa, FL, United States

**Keywords:** community gardens, COVID-19, food insecurity, food sovereignty, food supply chains, resilience, health

## Abstract

For quite some time, food systems scholars, public health workers, and food justice activists have recognized structural problems in the global food system that can cause food insecurity and inequitable access to nutritious foods. The COVID-19 pandemic and the accompanying disruption in food supply chains (FSCs) exposed these problems and raised questions about how community gardening and urban agriculture might offer some solutions. In this article, we examine the effects of the pandemic on the operations of community gardens in Central Florida and the attempts of these gardens to address the disruption in food supplies in their local communities. We do so by using data drawn from two research strategies employed by the members of University of South Florida’s Urban Food Sovereignty Group: (1) a survey conducted in 2021 of 45 leaders of community gardens throughout the Central Florida region; and (2) participation in the meetings and activities of a coalition of community gardens based in Tampa, Florida, from 2019 to 2022. Results reveal that although most community gardens in Central Florida were forced to change their routines in various ways (e.g., limiting the number of workers or volunteers who could work at the garden at the same time, or delivering workshops and education programs online instead of in-person), some gardens were able to maintain their regular operations and even reported increases in their membership or participation during the pandemic. Many community garden leaders also recognized the positive effect that community gardening seemed to have on their gardeners’ mental health, noting that their gardens became places of refuge for gardeners who sought safe, outdoor spaces, to relieve stress and interact with their neighbors. These same leaders also observed the effect of the pandemic on residents’ access to quality food, and intensified their efforts to provide more fresh food to pantries and traditional emergency food outlets. Survey respondents also shared important lessons they learned from the pandemic by suggesting that community gardens establish clear emergency protocols, use existing resources more efficiently, continue their educational programs, and strengthen their communications and cooperation with other gardens and actors in local food supply chains in order to ensure food security. We conclude by suggesting that one of the best ways that local communities can avert future food crises and strengthen their resilience is to root themselves more firmly in the principles of food sovereignty so they can sustain themselves when commercial FSCs are disrupted.

## Introduction

The increasing interest in community gardening among policy makers and community organizers in recent decades has sparked a number of research studies that examine community gardening management and outcomes across a variety of settings. These studies reveal that more and more communities across the world are establishing community gardens, and they are doing so with the intention of fostering individual and community well-being (1). As these studies report, community gardening offers a number of benefits to individuals and communities, including access to cheaper, healthier, and fresher foods, increased awareness of the possibility of growing one’s own food, skill-building and increased physical activity, enhanced mental health and social–emotional development, and increased community cohesion and resilience among fellow gardeners and those in their social networks (2–4). At the same time, community gardens experience a variety of routine challenges, ranging from insufficient participation by members of the community to insufficient funding, land, and materials (1).

In just the last few years, new research has emerged about how community gardens have responded to disruptions in food supply chains (FSCs) and heightened food insecurity caused by the COVID-19 pandemic in both the global north and the south. These studies reveal some of the new challenges posed to community gardens by the pandemic in such diverse settings as Canada (5, 6), China (7–9), France (10), Germany (11, 12), Italy (13, 14), Poland (10), the United Kingdom (15), the United States (2, 16), Australia (11, 17, 18), Brazil (19), the Philippines and Senegal (20). In general, these studies highlight the important role that community gardens can play in mitigating disruptions in FSCs, alleviating food insecurity, and increasing a variety of non-material benefits – what the Food and Agriculture Organization (FAO) labels “cultural services.”^1^ These cultural services include increasing self-reliance, providing access to open-air, socially distanced, and healthy environments, improving physical, mental, and spiritual health, and strengthening community resilience [see (21)]. At the same time, they reveal how the pandemic intensified already existing challenges, such as the shortage of volunteers, technical assistance, and government support.

This study seeks to add to our understanding of the operations and responses of community gardens in the aftermath of the outbreak of COVID-19 pandemic by looking specifically at how community gardens in the Central Florida area have responded to the challenges created by the pandemic. By focusing on the community gardens in Central Florida, our approach is neither to spotlight one particular garden, as many case studies do, nor to survey community gardens across the entire nation, as Drake and Lawson (1) have done in their survey of community gardens throughout the U.S. and Canada. Rather, drawing on data gathered from fieldwork among a coalition of community gardens based in the Tampa Bay area and a survey of the leaders of community gardens throughout Central Florida, we seek to understand what effect, if any, the COVID-19 crisis has had on these community gardens and the populations they serve. By doing so, we hope to illustrate the ways in which the responses of these gardens to the challenges posed by the pandemic are similar to the responses of gardens in other settings. Finally, we seek to learn from the community garden leaders themselves how they think their gardens might respond more effectively if faced with another crisis like COVID-19 in the future. Although the results we report here should not be extrapolated to all community gardens in other settings, by placing our results in the context of the emerging literature based on studies in other settings, we can highlight the similarities and differences.

## Review of the literature

The emerging literature focusing on COVID-19 and community gardening reveals that scholars are beginning to think differently about how more localized models of food production can mitigate food insecurity during periods of emergency food shortages (22, 23). The massive disruption in food supply chains revealed the precarity of our global food distribution system and its inability to effectively keep grocery stores stocked. The pandemic served as a moment to reflect on our connection to the food we eat, our physical proximity to our food sources, where and how it is produced, and the process involved in its distribution (24).

This emerging literature also reveals that community gardening can be an especially important resource for sustaining and promoting physical and mental health during disruptions of community life caused by measures of social distancing (13, 25). The COVID-19 pandemic limited people’s mobility and access to indoor community spaces, and gardens became an important option for those seeking to engage in safe physical and social activities. By offering this option, community gardening provided mental solace for those feeling confined to their homes during the early stages of the pandemic. Scoping review of the impact of gardening on health and well-being finds that gardening can potentially serve as a non-medical intervention for mitigating chronic diseases such as cardiovascular disease, chronic respiratory disorders, and cancer (3). Part of thinking about health and well-being beyond COVID-19 is understanding how food insecurity is more than just a lack of sufficient caloric intake, but rather includes a more holistic understanding of our physical, mental, and environmental health. This reinforces a variety of studies which illustrate how green spaces help connect urban citizens to nature in ways that can reduce anxiety, promote physical and mental health, and connect people with their natural surroundings (4, 5, 12, 14, 26).

As both the early and more recent research on community gardening reveals, community gardening is seldom simply about the production of food. Community gardening has a variety of positive outcomes for individual community gardeners, their social networks, and the larger communities of which they are a part. In addition to the direct positive outcomes highlighted above, community gardening can strengthen communities by fostering civic engagement, providing food security to the poor and homeless, encouraging or resisting gentrification, and promoting the values of food sovereignty (27). As previous research has revealed [e.g., (1)], the priority given to one or more of these goals depends on the characteristics of particular gardens – including their size, leadership, networks, funding – and the local context in which the garden resides – including the interests and ambitions of the participants, the availability of land, and the needs of the local community.

In the research presented here, we examine the operations and responses of community gardens in Central Florida in the aftermath of the outbreak of COVID-19 by surveying the leaders of 45 community gardens throughout Central Florida. By comparing our findings to those reported in recent studies of community gardens in other contexts, we seek to illustrate how the community gardens in Central Florida responded to the pandemic in ways that are similar to the responses of gardens in other settings. By placing our results in the context of the emerging literature based on studies in other settings, we can help to establish the generalizability of findings across numerous settings.

## Research methods

### Data

In order to identify the community gardens operating in the Central Florida area in 2021, we drew on three primary sources – (1) the website of the American Community Gardening Association which lists the gardens that are members of their Association, (2) the listing of gardens that were part of the Coalition for Community Gardens of Hillsborough County, FL, and (3) information we received from key informants and respondents as we were conducting the research during the summer and fall of 2021.^2^ We believe that by drawing on these various sources we have arrived at a nearly comprehensive listing (79 in total) of the publicly identifiable community gardens in the Central Florida area, an area which extends across 13 counties in a region of Central Florida between the Gulf of Mexico and the Atlantic Ocean. Of the 79 community gardens identified, the overwhelming majority (62 out of 79) are located in the three counties of the Tampa Bay region – Hillsborough, Pinellas, and Sarasota. Thirty-one (31) gardens are in Hillsborough County, 23 in Pinellas County, and 8 in Sarasota County. The remaining 17 community gardens are located across the other 10 counties, including 4 in Pasco County, 2 each in Orange and St. Johns Counties, and 1 garden each in Brevard, Hernando, Lake, Manatee, Marion, Okaloosa, Osceola, Palm Beach, and Polk Counties.

Using this list of 79 community gardens as our sample frame, we obtained contact information for the leaders or managers of all 79 community gardens and sent them an email in which we invited them to complete an online survey questionnaire which we composed and administered using Qualtrics. To ensure that the questions in our survey were impartial and our respondents’ answers were unbiased, we used clear and neutral wording, avoided the use of any leading or biased questions, incorporated both Likert scales and open-ended questions, and guaranteed the anonymity of our respondents. We also had our survey instrument reviewed by the University of South Florida’s Institutional Review Board (#001458), two independent experts, and then pilot tested the survey to get feedback on potential bias or confusing wording. Finally, we conducted a debriefing session to review the feedback to our pilot test and determine any final changes to the survey instrument.^3^

In order to ensure fullest participation in the survey, we extended the period of completion over a six-month period, from July to December, 2021, and sent multiple follow-up reminders. Forty-five (45) of the 79 community gardens we contacted responded to our request to complete the survey, yielding a response rate of 57%.

Although survey research may sometimes overlook important factors in local contexts that are best captured through in-depth case studies of individual gardens, we believe we were able to capture the characteristics of the local contexts by using specially designed survey questions that asked respondents about how their gardens responded to local challenges created by the COVID-19 pandemic. Furthermore, because the respondents to our survey are the leaders or managers of the community gardens, they are highly aware of the communities in which their gardens operate, and some of the open-ended questions on the survey asked them to reflect on how the community context shaped their gardens’ experiences during the pandemic. Like Drake and Lawson’s (1) survey of community gardens in North America, we asked respondents about their garden’s location and service areas, partnerships, benefits, and challenges.^4^

Although the data obtained from this survey are the primary basis of our analysis, we are also informed by: our observations between 2019 and 2022 of the meetings and activities of the Coalition for Community Gardens of Hillsborough County, which is based in Tampa, Florida; our participation in the University of South Florida’s Urban Food Sovereignty Group since its inception in 2019; and our involvement in a number of the community gardens that are affiliated with these entities. Both authors have been active members in these groups since 2019, and the lead author has been an active participant in an initiative called the “Healthy 22nd Street Garden Initiative” in East Tampa.

### Models of community gardens

Previous research on community gardens in North America and our own observation of community gardens in the Central Florida area have revealed that community gardens tend to employ one of the following three models: One model is a garden that features individually rented and maintained garden plots within a communally maintained garden space. Although the overall garden site is collectively operated, each gardener is assigned a plot within which they grow and harvest their own fruits and vegetables. A second model is to have a single, communal garden in which everyone participates in growing and harvesting the communally shared fruits and vegetables. This model, instead of having individually assigned garden plots, operates as a truly communal endeavor in which all members work collectively, often deciding collectively what crops to grow and how to divide and distribute the harvests. In some cases, the members of these communal gardens decide to split the harvests based on the number of hours each member has volunteered to the maintenance of the garden. In other cases, they may decide to donate their harvests to local food pantries or soup kitchens. A third model, what might be called a hybrid model, is one that features both individually rented plots and communal plots. In this third model, each gardener decides which type of garden space they prefer to maintain based on their resources, desired level of commitment, and desired form of interaction with other gardeners.

Of the 45 community gardens represented in our sample, 7 (or 16%) reported that they had adopted the first model in which separate garden plots were individually rented and maintained throughout the space, 19 (or 42%) stated that they employed the communal model in which gardening was done communally on a common plot of land, 16 (or 36%) reported that they had adopted a hybrid model in which both individual and communal plots were available for gardeners, and the remaining respondents reported using some other type of model, such as a “school garden” or a “pantry farm,” which can be regarded as similar to the communal model.

## Results

In order to evaluate the effect that COVID-19 had on the community gardens we studied in Central Florida, we asked survey respondents a series of questions, including: “What effect, if any, did the COVID-19 crisis have on your community garden or the populations it serves? Were you aware, for instance, of any situations of food insecurity?” “How did you or the leaders of your community garden respond, if at all, to the challenges created by the COVID-19 crisis?” “In the face of another crisis like COVID-19, how might your community garden -- or other community gardens in general -- respond to better ensure the health and security of the community?” Responses to these questions reveal that the COVID-19 pandemic affected the community gardens in our sample in variety of ways, some neutral or positive, and some negative.

### Impact of COVID-19 on community garden participation

#### Neutral or positive effects on participation

Interestingly, according to the community garden leaders we surveyed, many gardens remained open during the initial months of COVID-19. The leaders of 14 (or 31%) of the 45 gardens represented in our survey reported no significant change to the everyday operations of their gardens. Those who reported no significant change in their operations often stated that the most significant difference was the need to practice social distancing or to wear a mask.

Five (5) of the 45 community garden leaders in the survey (or 11%) reported that membership or participation in their garden actually increased during the pandemic. These garden leaders perceived that the gardeners in their community gardens found the outdoor setting of gardens as one of the only options for safe activity. As one respondent indicated: “COVID-19 increased usage of community garden in that it gave people an outdoor outlet.” Another noted, “our garden was the only place that people felt safe coming to. It was a refuge during a very difficult and stressful time and I’m grateful my gardeners felt a sense of security and comfort during an unprecedented time.” These responses amplify one of the commonly seen benefits of community gardening in general, with or without a pandemic; namely that community gardening provides an opportunity for gardeners to relieve stress and enjoy a quiet place to reflect and unwind (2). One respondent stated that gardening is a way to “relieve the everyday stresses by getting out in the fresh air, digging in the dirt, and reaping the therapeutic value of learning and creating.”

Another community garden leader reported observing a surge in interest in community gardening among the members of the local community, which resulted in an increase in membership and participation at the garden. This community garden leader attributed this to people’s search for a sense of community in the midst of the pandemic: “We kept our garden going, and we have had a boom in membership sign-ups as people look for community and something to do away from their computers.” In a similar vein, other leaders of these community gardens highlighted how grateful community members were for the gardens remaining open. Although, as we will discuss below, gardens hosted by public schools were forced to shut down or restrict programs, one respondent reported that at their garden “more people [who were not previously affiliated with the garden] saw it [the garden] and asked about what the different vegetables were, and I got to talk to more people about how to cook things, what they tasted like, why they were healthy, etc.” Clearly, in this case, the pandemic seemed to stimulate interest in gardening among community residents who had previously not been interested.

These findings are similar to those reported in other studies. After the outbreak of the COVID-19 pandemic, as public health officials cautioned people about the risks of infection in indoor spaces with poor air circulation, people were afraid to engage in activities in indoor spaces and resorted to outdoor activities to fulfill their social needs and break the monotony of remaining in the confines of their homes. The outdoors in general, and community gardens in particular, became safe spaces during the pandemic, “with gardens serving as a place for families and friends to access fresh air, exercise, and enjoy the mental health benefits of green space” [(2), p. 8].

A leader of one of our community gardens – one that also functions as an urban farm - described how the pandemic had at least a couple positive effects on its operations.^5^ He reported that the pandemic provided the opportunity for their garden to expand their land and purchase a new building that will be used to build a grocery co-op and café at the site of the new garden. Moreover, the garden operator highlighted increased use among customers of their Electronic Benefits Transfer (EBT) cards and the resources of the Supplemental Nutrition Assistance Program (SNAP) to purchase fresh produce from the garden, something he attributed to the increased reliance on public assistance among those who found themselves unemployed as a result of the pandemic.

#### Negative effects on participation

Despite many garden leaders reporting either little to no change in garden operations or an increase in participation during the pandemic, other gardeners expressed contrasting sentiments. Eighteen (or 40%) of the 45 respondents stated that COVID-19 had some type of negative impact on the regular operations of their gardens. Consistent with other studies that show closures of gardens early in the pandemic [e.g., (21)], several of the gardens in our survey reported closures. Gardens that were affiliated with public schools or some other type of government entity, for instance, were forced to close because of the legislatively mandated shutdowns. This resulted in lack of access to garden spaces and materials, and the suspension of educational programs connected with these gardens, especially the school gardens.

Some gardens reported that they only needed to shut down temporarily or for a short period of time. In the case of partial closures for example, one garden reduced staff to a skeleton crew and limited the number of gardeners who could work in the garden. Another garden reported that it kept a core team producing food throughout the pandemic. In several cases, garden leaders implemented creative strategies, such as alternating weekend planting, harvesting, and weeding responsibilities. One garden extended their hours of operation in order to practice social distancing yet allowed for continued participation. Six of the 45 garden leaders (or 13%) specifically highlighted the decline or loss of volunteers, citing city closure protocols or gardeners’ fear of exposure to COVID-19 as reasons for their staying home. None of the garden leaders reported a severe negative impact due to the loss of volunteers, despite the maneuvering that some gardens had to do to stay open.

### Perceived food insecurity and the gardens’ responses

Understanding the impact of the pandemic on food insecurity in the communities surrounding the gardens was a little more difficult to assess. Respondents were asked: “Were you aware of any situations of food insecurity? How did you or the leaders of your community garden respond, if at all, to the challenges created by the COVID-19 crisis?”

Of the 45 community garden leaders surveyed, 12 (or 27%) reported some degree of awareness of increased food insecurity in their communities, while 4 (or 8%) indicated no perceived awareness of food insecurity. Many of the garden leaders reported that their gardens were giving fresh produce directly to community members. One particularly extreme example of food insecurity was described by a respondent who stated: “We had more hungry/desperate individuals approach us for food and [they] were willing to take anything that was available, even with no way to cook or prepare it.” Several of the gardens identified their neighborhoods as already food insecure and, as such, COVID-19 only exacerbated an existing problem. Further information about garden leaders’ perceptions of food insecurity will be discussed below in the responses to how garden leaders envision preparing for future food system emergencies.

Although not all of the gardens in our survey are considered by their leaders to be primarily focused on community food production, the pandemic did result in efforts to produce more food for the community. In the early stages of the pandemic, many of the garden leaders recognized the potential long-term implications of COVID-19, and sought to increase food production itself. As one garden leader noted, “We started food distribution of some of the produce being grown, but demand soon outstripped production.” Nine (or 20%) of the 45 garden leaders indicated that they attempted to produce more food, either through growing more vegetables or providing more resources (human labor, money, and time) to charity or other food assistance efforts. One leader noted that their garden planted more collard greens, a particularly popular vegetable to grow in central and southern Florida. Along these lines, another garden leader commented on the dual need to provide both food and comfort to community members who were anxious or frightened by the unknowns during the initial months of the pandemic. With respect the theme of reducing anxiety and growing culturally important foods, the leader of one garden noted that they “assuage their [community members’] fears instantly by the sharing of local, nutritious, wild urban foods and medicine (weeds). We also share novel, very easily grown (near indestructable [*sic*]) “superfood” species that are staples in third world countries and that thrive in Florida’s hot and humid climate.” Lastly, one of the greatest examples of the motivation to produce more food was the creation of a new garden, the 42nd Street Garden of Miracles in Tampa, during the height of the pandemic.

Perceived food insecurity was an underlying theme that motivated many of the garden leaders to maintain operations to the best of their ability. As mentioned, nine (or 20%) of the 45 garden leaders specifically mentioned the attempt to produce more food due to increasing food insecurity. In many cases, these garden leaders used creative strategies to provide food to the community. For example, one garden leader reported: “We identified families who were in need of food, created food drives and had staff to deliver food to houses or to community locations, and used social media to inform residents where to pick up food.” This proactive approach signaled an underlying sense of initiative that community garden leaders expressed. Along these lines, another garden leader noted: “We used it [the pandemic] as a driver to accelerate our work by creating more opportunities for the community to get involved.” Even as some gardens needed to shut down or reduce their open hours, garden leaders adapted in order to continue operations to the best of their ability.

Many garden leaders recognized the need for creative and multifaceted approaches to address perceived food insecurity. Efforts involved growing more food, utilizing existing resources more efficiently, and working cooperatively with other garden leaders and food production and distribution agencies. Summing up this sentiment, one garden leader conveyed that “Leaders decided that the community needed assistance now more than ever, and despite the challenges of COVID-19, found innovative ways to distribute food, connect with others, and return to work/neighborhood activities as soon as possible.” One community garden leader, in describing the non-growing activities of their garden, noted that after the outbreak of the pandemic, gardeners spent “more time collecting nonperishable items.” Another garden leader described how they “reached out to other community gardens to donate any excess harvests they had -- no matter how small -- to get more fresh produce to our clients.” Given reduced access to physical gardens and food distribution outlets, cooperating with local food banks was another strategy for garden leaders. As one leader noted: “We shared other resources for acquiring food from local food banks.”

### Adoption of COVID-19 safety measures

In responding to the question “How did you or the leaders of your community garden respond, if at all, to the challenges created by the COVID-19 crisis?,” some of the community garden leaders described how they adopted the necessary COVID-19 safety measures, just like other organizations, in order to maintain their daily operations.

Fifteen (or 33%) of the 45 garden leaders in our survey directly expressed the need to address the pandemic, at least during its early stages, through precautionary tactics such as canceling operations, encouraging social distancing, wearing gloves and masks, and regular sanitation practices. Five (or 11%) of the garden leaders reported the need to close or cancel standard operations in the initial stages of the pandemic. In the face of closures or canceled community events and classes, five leaders (or 11%) reported moving member meetings to a virtual format, and 10 (or 22%) mentioned the need to sanitize, wear masks, and socially distance. Two garden leaders specifically mentioned the desire to create comfortable spaces for people to gather and continue to learn about garden activities.

### Lessons from COVID-19

Although we found that the community gardens in Central Florida were able to survive the worst months of COVID-19 infections and shut-downs, many garden leaders we surveyed expressed concern about how community gardens might respond more effectively to similar crises in the future. We asked respondents: “In the face of another crisis like COVID-19, how might your community garden -- or other community gardens in general -- respond to better ensure the health and security of the community?” The majority of respondents acknowledged that community gardens could be better prepared to respond to future crises not only to ensure the health and safety of their gardeners, but also to ensure access to affordable, nutritious, and fresh food when disruptions in commercial food supply chains cause local food shortages.

Among the primary ways our community garden leaders believe their community gardens could prepare for future crises are: (1) to be ready to provide whatever public health safety measures (e.g., sanitizers, masks, strategies for social distancing) are called for in response to the public health crisis; (2) to increase education programs so community residents are aware of their ability to grown their own food when commercial suppliers experience shortages; (3) to adopt more efficient modes of communication and collaboration between individual gardeners and community gardens in each region to ensure coordinated food production and distribution; and (4) to develop coordinated strategies to increase local food production as an alternative to commercial food suppliers.

The COVID-19 pandemic dramatically affected the way people interact socially, and community garden leaders, like many other industry leaders, were forced to provide safe and sanitary working conditions. Eight (or 18%) of the 45 community garden leaders we surveyed specifically mentioned that they felt they needed to be better prepared to provide sanitary working conditions and to encourage community members to take precautions such as social distancing, wearing gloves, and washing hands. These garden leaders also recognized that they play an important role in modeling these health care precautions, as one garden leader acknowledged by commenting: “Following normal health cleanliness requirements is generally effective if they were practiced by all, and especially by the leaders, people like that.” At the same time, other garden leaders believed that these safety measures were the responsibility of everyone who participated in the gardens, and as long as everyone followed public health protocols, garden managers would not need to make special efforts to monitor adherence to those protocols. For instance, one garden leader mentioned, “I’m not sure we would do anything differently. Gardeners followed the safety guidelines.” Another garden leader stated: “I think we might react in a similar manner, asking gardeners to wear gloves and masks in our garden, for the comfort of all of our gardeners. This allowed us to continue growing food and tending our little oasis.”

Five (or 11%) of the 45 garden leaders surveyed voiced the desire to host more educational programs that would encourage and enable community residents to grow their own food, provide their own food security, and/or donate the harvests of community gardens to food pantries and soup kitchens. One garden leader, when asked “how might your community garden…respond to better ensure the health and security of the community?,” replied by connecting themes of education, food security, and collaboration. In responding to this question, he relayed the following grand vision:

“First of all, by creating a real tangible database of gardening members who are willing to share their harvest, knowledge, and ideas. Second by hosting large scale tours monthly at various gardens to show the communities what exists in their region. Lastly and most importantly, I hope that because of COVID-19, more gardens have been created so our neighborhoods realize these resources exist.”

This idea of tackling food insecurity through more holistic strategies of knowledge-sharing, education, communication, and collaboration was also expressed by other garden leaders.

The need for improved forms communication and collaboration among participants in each garden, between gardens, or between gardens and other organizations (e.g., schools or food distributors) was raised by seven (or 15%) of the community garden leaders. Within this group, two garden leaders mentioned the need to develop or conduct more virtual classes so those who are hesitant to engage in face-to-face interactions can continue to learn about gardening, share knowledge, and provide each other with advice. Two leaders also mentioned the desire to improve communications and relationships with local schools, noting that “Better communication with the schools [is necessary] so that we can teach more classes in case this happens again.” Some community garden leaders also recognized that improved communication and collaboration among community garden leaders and others in the food supply chain was necessary to respond more effectively to food supply crises in the future. One Native American farmer-organizer noted, for instance, “It would be good if all community gardens communicated with one another and worked together to make a wider variety of food accessible to our different constituents.” Another leader stated, “We need to collaborate more with other farmers, purchase more from other farmers, increase our offerings to the public and community, more farm tours, classes, workshops and events.” Another leader stated that more collaboration was needed to develop a “strong local food system to increase community resilience with food security, community involvement, and the availability of more local nutrient-dense fresh food, vegetables, fruit, and protein.” According to another garden, developing a strong local food system will require creating better access to various food distribution points for their garden’s produce.

As these responses illustrate, the community garden leaders we surveyed recognize that major crises like COVID-19 can cause disruptions in commercial food supply chains that leave many communities without a sufficient supply of affordable, nutritious, fresh food. They also recognize that community gardens can serve as a critical supplier of affordable, nutritious, fresh foods to local communities, but that in order for them to respond most effectively in the face of future disruptions, they will need to strengthen their education programs, communication platforms, and collaborative relationships across a variety of domains in the local food system.

## Discussion

As Drake and Lawson (1) have shown based on their survey of community gardens in the United States and Canada, community gardens often focus on one or more of the following goals or outcomes: increasing access to healthy foods, promoting healthy eating habits, providing environmental education, and fostering community development. Seldom do community gardens focus on only one of these goals, and some scholars argue that food production goes “hand-in-hand” with the community development goals of gardens [(1), p. 243; see also (27–29)]. The community garden leaders we surveyed also identified these important, multifaceted functions of community gardens, but they also noted how critical these functions became in light of the challenges presented by the COVID-19 pandemic.

An important theme that emerged in our data is that community gardening was perceived by our respondents to provide access to open-air, socially distanced environments where gardeners could maintain their physical, mental, and spiritual health, while simultaneously producing their own food. These findings are similar to those reported in other studies (30). After the outbreak of the COVID-19 pandemic, as public health officials cautioned people about the risks of infection in indoor spaces with poor air circulation, people were often afraid to engage in indoor activities and resorted to outdoor activities to fulfill their social needs and break the monotony of remaining in the confines of their homes. The outdoors in general, and community gardens in particular, became safe spaces during the pandemic, “with gardens serving as a place for families and friends to access fresh air, exercise, and enjoy the mental health benefits of green space” [(2), p. 8]. Community gardens became sites of refuge amidst the stresses and anxieties caused by the pandemic.

In light of these positive effects of community gardening on the physical, mental, and spiritual wellbeing of the gardeners, it is unfortunate that government-sponsored gardens, such as those affiliated with public schools, were forced to shut down during the pandemic. While these shutdowns were understandable during the early stages of the pandemic when it was still uncertain how the virus was spreading, if these gardens had been permitted to re-open immediately after scientists learned that the primary risk of transmission was in indoor spaces, then more people, especially young people, could have enjoyed the salutary effects of community gardening throughout the pandemic. In the future, if our society is plagued by a similar type of virus whose transmission is primarily confined to indoor spaces, public health officials may wish to promote more forcefully the value of community gardening not only for providing food security during the disruption in commercial food supply chains, but also for protecting the physical, mental, and spiritual health of the larger population, particularly those who are most vulnerable to the negative consequences of social isolation. Because of the beneficial effects of gardening, community gardens, just like grocery stores and health care clinics, should qualify as providing “essential services” for ensuring the vitality of the community during times of crisis. Some national food policy councils have begun to recognize this and have called for government officials to classify community gardens as “essential services.”

Another theme in the responses of the community garden leaders in Central Florida was the need to be better prepared to address food insecurity in the face of similar system-wide food crises in the future. Respondents noted the need for more educational programs, whether in connection with local schools, community gardens, or urban farms, or through collaborative community initiatives designed to raise awareness of the value of community gardening to mitigate the negative consequences of disruptions in commercial food supply chains (FSCs).

One such example that emerged after the early stages of the pandemic was the Healthy 22nd Street Garden Initiative in East Tampa. This initiative involved the creation of a neighborhood demonstration garden that served and continues to serve as the central meeting place for conducting educational workshops on a variety of food production opportunities – planting, growing, harvesting, and seed saving; canning and food preservation; and culinary demonstrations (31, 32). In this project, community members are supplied with an innovative “barrel garden” – a food-grade barrel fabricated to be a self-watering home-garden. Gardeners are able to use these barrels to grow their own vegetables in small spaces and with limited amounts of water. Similar to the findings of Buechler and Mansaray (2), home gardeners in the Healthy 22nd Street Garden Initiative took matters into their own hands during the pandemic by growing their own healthy foods rather than relying exclusively on foods available through commercial grocers.

Another educational initiative in the Central Florida region is seen in the programs offered by a local community garden/urban farm in South St. Petersburg called the St. Pete Youth Farm. Through gardening and education programs, the St. Pete Youth Farm provides a welcoming place where young people can “learn everything from growing their own food to financial literacy.”^6^ Located in an under-resourced neighborhood where youth are at risk of poverty, food insecurity, and various other social inequities, the St. Pete Youth Farm strives to amplify the existing strengths of the community by empowering youth to develop sustainable food sources while simultaneously encouraging leadership, entrepreneurship, and career readiness among its participants (33). This potential for community gardens, or other urban food production projects such as urban farms, to raise awareness, educate, and foster future leaders was captured in the statement of one of our respondents who noted, “most importantly I hope that because of COVID-19 more gardens have been created so our neighborhoods realize these resources exist.” Today the St. Pete Youth Farm continues to flourish and has added a green house, and an aquaponics system that grows vegetables and produces tilapia for community consumption. Not only has it survived the early years of the pandemic but it has also weathered recent natural disasters, including hurricanes Ian and Nicole. The St. Pete Youth Farm, like similar small-scale urban production initiatives in other settings, represents how gardening and education can be combined to strengthen multiple aspects of community life (2).

The examples of the Healthy 22nd Street Garden Initiative in East Tampa and the St. Pete Youth Farm in South St. Petersburg demonstrate that the value of community gardening extends beyond the provision of affordable, nutritious, fresh food during disruptions in commercial food supply chains. Community gardens also promote gardeners’ overall health and well-being, their social–emotional development, skill-building, entrepreneurship, and leadership, which ultimately advance the cohesion and resilience of the community. Initiatives like these represent innovative and effective models for providing food security and enhancing community well-being and cohesion, which are critically necessary during a global pandemic.

In many ways, the COVID-19 pandemic, by drawing attention to the vulnerabilities of commercial FCSs, has raised awareness among many of our respondents of the value of pursing food sovereignty as a means to achieve food security (34).^7^ Although our respondents did not use the term “food sovereignty” to describe how their gardens could be better prepared to respond to future food crises, they recognized the value of empowering local communities to produce and distribute their own locally grown food. They recognized the value of increased communication and cooperation among local growers, community gardens, and local distribution points. They also recognized that each community needs to develop a coordinated strategy among agents within their own community to respond to the shortcomings of the industrialized food system.^8^

One of the chief strengths of our analysis is that it has confirmed the various positive outcomes of community gardening as perceived by our survey respondents. However, our study also has some limitations. Because of our limited resources and the restrictions of the pandemic, we were unable to gather what might be regarded as more objectively verifiable measures of the outcomes of the community gardening initiatives we studied. We were not, for instance, able to extend the scope of our survey beyond the community garden leaders themselves to also include the numerous gardeners who remained active in the gardens through the time of the pandemic, or the various organizations that benefitted from the harvests of these gardens, including local food banks, food pantries, and soup kitchens. Nor were we able to collect objective data on the size of each garden’s harvests or the portion of their harvests that were given to local food banks, pantries, and kitchens. Having these additional data would have allowed us to corroborate the perceptions and claims of the community garden leaders, particularly their claim about increased food production during the pandemic in order to offset the disruption in the commercial FCSs. Hopefully future research can include these additional kinds of measures not only to verify the perceptions and reports of the community garden leaders, but also to evaluate more precisely the extent to which community gardens can help reduce food insecurity in the communities in which they are located.^9^

In short, while many of the community garden leaders we surveyed were certainly convinced that their gardens needed to increase food production to help offset reductions in food supplies caused by disruptions in commercial FSCs, the only way to know this definitively would be to examine with more direct measures the extent of the disruption in FSCs in the Central Florida area and the extent to which community gardens were able to fill the void. Collecting these additional data will be necessary in order to accurately quantify the extent to which community gardens and local food systems can adequately meet the needs of local consumers when our existing commercial FSCs are disrupted. This will be especially important in determining the extent to which local growers can meet the needs of vulnerable populations that rely on the food banks, food pantries, and soup kitchens, which often rely on the excess or leftover produce donated by commercial grocers. Our study would have been able to address these topics more definitively if we had been able to collect data across the duration of the pandemic on fluctuations in food supplies among local grocers, food banks, food pantries, and soup kitchens, as well as fluctuations in food harvests among the community gardens in our survey. By addressing these issues, researchers would be able to make more accurate assessments of whether community gardening is a viable substitute for commercial FSCs. If community gardening is not currently a viable substitute for commercial FSCs in the event of a disaster or other public health crisis such as a pandemic, then what might community gardens and the local officials who support them do to make them better equipped to respond? Our survey respondents hint at the answer to this question, but they do not answer it definitively.

## Conclusion

On a global level, the COVID-19 pandemic has raised awareness of the potential for community gardening and urban agriculture to meet a variety of individual-and community-level needs when commercial food supply chains (FSCs) are disrupted by emergencies. In order to examine the ways in which community gardens throughout Central Florida responded to the pandemic, we surveyed community garden leaders to ask them about their perceptions of the effect of the pandemic on their own gardens and the larger communities in which they reside.

Our results revealed that although most community gardens in Central Florida were forced to change their routines in various ways as a result of the pandemic, some gardens were able to maintain their regular operations and even reported increases in their membership or participation during the pandemic. Many community garden leaders, recognizing the detrimental effect of the pandemic on the community’s mental health, noted that their gardens became a place of refuge for gardeners who sought safe, outdoor spaces, to relieve stress and safely interact with their neighbors. These same leaders also observed the effect of the pandemic on residents’ access to quality food, and intensified their efforts to provide more fresh food to local food pantries and traditional emergency food outlets.

Our survey respondents also shared important lessons they learned from the pandemic so they can be better prepared for future FSC crises. They suggested that community gardens establish clear emergency protocols, use existing resources more efficiently, continue their education programs, strengthen their communications with other actors in local food supply chains, and work cooperatively with other community gardens and food production and distribution agencies to ensure an adequate supply of food for local residents.

Because these recommendations of the community garden leaders align with the some of the goals of what has come to be known as the “food sovereignty movement,” particularly with respect to empowering local communities to produce and distribute their own locally grown food, our data support the potential benefits to local communities of developing coordinated strategies among agents within their own communities to respond to the shortcomings of the industrialized food system (37–43). Although our data cannot estimate the extent to which community gardens can effectively meet consumers’ needs during future disruptions in FSCs, they do confirm the perception among our survey respondents of the numerous salutary benefits of community gardening during times of crisis. Local public officials, we believe, would be wise to recognize the potential for community gardening and urban agriculture to meet a variety of individual-and community-level needs during times of crisis, as well as their potential to ensure food security through unexpected disruptions in commercial food supply chains (44).

## Data availability statement

The raw data supporting the conclusions of this article will be made available by the authors, without undue reservation.

## Ethics statement

The studies involving human participants were reviewed and approved by USF Institutional Review Board. Written informed consent for participation was not required for this study in accordance with the national legislation and the institutional requirements.

## Author contributions

WS study design, correspondence and application for Institutional Review, data analysis, and writing. JC study design, data analysis, and writing. All authors contributed to the article and approved the submitted version.

## Funding

This sudy was supported by the University of South Florida.

## Conflict of interest

The authors declare that the research was conducted in the absence of any commercial or financial relationships that could be construed as a potential conflict of interest.

## Publisher’s note

All claims expressed in this article are solely those of the authors and do not necessarily represent those of their affiliated organizations, or those of the publisher, the editors and the reviewers. Any product that may be evaluated in this article, or claim that may be made by its manufacturer, is not guaranteed or endorsed by the publisher.

## Footnotes

^1^https://www.fao.org/ecosystem-services-biodiversity/background/cultural-services/en/, retrieved on 5/24/2023.

^2^Using the “find a garden” function on the website of the American Community Gardening Association, we identified 46 gardens in the Central Florida area which are members of this association. Using the listing of gardens which are part of the Hillsborough County Coalition of Community Gardens, we identified an additional 13 gardens in that county alone. The remaining 20 gardens were identified from information provided by key informants and respondents during the course of our research. These search methods resulted in a sample frame of 79 community gardens across thirteen counties in Central Florida.

^3^We also collected demographic information about the survey leaders themselves. The majority of community garden leaders were 50 years or older, female, white, and had attained at least a bachelors degree. For this particular article, which focuses on the effect of the pandemic on community gardens, we do not have a theoretical rational for believing that the personal background characteristics of our respondents would have an influence on how the community gardens experienced or responded to the pandemic.

^4^Interestingly, in describing the “unit of analysis” in their survey of community gardens in North America, Drake and Lawson (1) state: “Organizations” – groups that identify as supporting community gardening – are our unit of analysis, and community garden practice is the object of study. We did not distinguish between non-governmental organizations, government agencies, or informal groups in part because there is considerable overlap between organizational typologies. As a result, the organizations that Drake and Lawson surveyed varied in size, with some managing only one community garden, others managing 2–3 gardens, some managing 4–30 gardens, and others managing 31 or more community gardens. While the same is true of the organizations that manage community gardens in Central Florida, we specifically aimed to reach the leaders and managers that oversaw the operations of each specific community garden in our sampling frame.

^5^Although this particular community garden includes an urban farm, it clearly falls under the communal model of community gardens described above.

^6^See https://stpeteyouthfarm.org/about, retrieved April 5, 2023.

^7^The food sovereignty movement has its origins in the (35) Neyelani Declaration on food sovereignty, which recognizes food as a basic human right and the rights of peoples to define their own food and agricultural systems [Declaration of Nyéléni (35)].

^8^Schanbacher and Gray (36) provide evidence that community gardens and urban farms can be effective in sharing resources with existing local food distribution outlets, such as food banks, religious organizations, or other charitable organizations.

^9^Perhaps the most challenging issue that community gardens and urban farms might face during future disruptions in local FSCs is their ability to scale to production levels needed for their populations. We believe this will require commitments that go beyond current measures used to ensure food security, many of which rely on the excess or leftover food provided by local grocers. It will require, in our opinion, a more holistic approach to food production and distribution that is grounded in the principles of food sovereignty.
